# Hydrodistillation-Based Essential Oil Extraction and Soda Pulping of Spent Hemp Biomass for Sustainable Fiber Production

**DOI:** 10.3390/molecules31030500

**Published:** 2026-01-31

**Authors:** Munmun Basak, Stephen C. Agwuncha, Sharmita Bera, Margaret Bloomquist, Jeanine Davis, Lucian Lucia, Lokendra Pal

**Affiliations:** 1 Department of Forest Biomaterials, North Carolina State University, Raleigh, NC 27695-8005, USA; 2 Department of Horticultural Science, North Carolina State University, 455 Research Drive, Mills River, NC 28759, USA

**Keywords:** floral hemp, spent biomass, essential oil, hydrodistillation, sustainable fibers

## Abstract

Hemp (*Cannabis sativa* L.) is increasingly valued not only for its fibers and seeds but also for essential oils derived from floral by-products. This study investigates the extraction of essential oils from three hemp floral varieties, Sour Space Candy, Suver Haze 3N, and Pinewalker 3N using hydrodistillation, a widely accepted and efficient method for isolating volatile compounds. The chemical composition and quantification of key volatiles, including α-pinene, β-myrcene, α-humulene, and α-terpineol, were analyzed using gas chromatography–mass spectrometry (GC–MS). In addition to oil extraction, the residual spent biomass was repurposed into pulp fibers using the soda pulping process. Fiber properties such as freeness, viscosity, kappa number, and fiber length were evaluated for papermaking applications. The essential oil yield ranged from 1.24% to 1.86% (*w*/*w*), and the spent fiber yield ranged from 37.07% to 55.23%. Handsheets prepared from blends of spent fibers and hemp hurd fibers exhibited tensile indices ranging from 21.87 to 34.98 N·m/g. This dual-valorization approach enhances the economic and environmental value of hemp cultivation, supports sustainable material development, and contributes to the broader adoption of bio-based alternatives.

## 1. Introduction

Hemp (*Cannabis sativa* L.) has gained global attention not only for its versatility in producing fiber, seeds, and biomass, but also for its unique phytocannabinoids [1]. Traditionally cultivated for its seeds and fiber across Asia, Canada, and parts of Europe, hemp is now recognized for its ability to produce niche products like essential oils [2,3]. Extracted from leaves and flowers after the separation of fibers, hemp essential oil represents a commercially viable product [4]. Utilizing these by-products to create valuable chemicals could further enhance the economic value of the crop.

The increase in hemp cultivation for essential oil extraction from floral parts and leaves has been driven by growing interest in the medicinal and therapeutic benefits of non-psychoactive cannabinoids, such as cannabidiol (CBD), cannabichromene (CBC), cannabidivarin (CBDV), and cannabigerol (CBG) [5]. Terpenes represent the largest group of chemical compounds found in hemp essential oil [6]. More than 100 terpenoids, including 58 monoterpenoids, 38 sesquiterpenoids, two triterpenoids, one diterpenoid and four other compounds, are found in hemp essential oil [7]. Terpenes contribute to the characteristic smell and flavor of various *C. sativa* strains, whereas phytocannabinoids are odorless [8]. The highest essential oil production is concentrated in the glandular trichomes of the unpollinated flowers of female hemp plants [9].

Terpene profiles in cannabis are characterized not only by their diversity, but also by pronounced differences in relative abundance and composition among cultivars. Volatile terpene fractions are typically dominated by monoterpenes, which can account for the majority of total terpene content, while sesquiterpenes contribute substantially to the heavier and less volatile fraction. Reported concentrations of total monoterpenes in floral tissues range from approximately 3.1 to 28.3 mg/g dry flower weight, whereas sesquiterpenes such as β-caryophyllene and α-humulene are commonly present at levels of 0.5–10.1 mg/g dry flower weight [10]. These quantitative differences result in cultivar-specific terpene profiles that are frequently used as chemotaxonomic markers [11]. However, terpene composition is highly sensitive to genotype and environment interactions, and cultivation conditions, plant maturity, mineral nutrition, light intensity, temperature, irrigation, and harvest timing can significantly alter the relative contribution of individual mono- and sesquiterpenes [12,13]. This variability highlights the importance of cultivar selection and controlled agronomic practices when evaluating terpene profiles in floral hemp.

Given the chemical diversity and variability of terpene profiles, the method chosen for essential oil extraction plays a critical role in determining both yield and composition. Various methods have been explored to extract essential oil from floral hemp. Among them, steam distillation [14], supercritical fluid extraction [15], and solvent extraction are most prevalent. Steam distillation is the most commonly used method for commercial extraction of essential oil, and it is believed that ~93% of essential oil is extracted by this method [16]. In one study, hemp essential oil was extracted via steam distillation at 130 °C from leaves and separated into five fractions based on volatility and boiling points [14]. The drawbacks of this process are that the yield of essential oil is low due to insufficient wetting of the biomass by steam, and also, the high amount of energy required to generate the steam [17].

In another study, essential oil extraction was carried out with a laboratory supercritical fluid extraction unit using carbon dioxide (CO_2_) as the solvent at a mild temperature range of 40–50 °C and pressure of 100–300 bar [15]. Efficient and effective, this process is not widely used commercially mainly due to high equipment investment costs and the requirement for highly trained personnel [18]. Solvent extraction is a conventional method of extracting essential oil by reflux solvents such as hexane, ethyl acetate, or ethanol, but it is time-consuming and requires high organic solvent consumption [19].

Another newly explored extraction is the microwave-assisted method, which minimizes processing time and increases the yield of essential oil, but may cause the breakdown of antioxidants due to microwave radiation [20].

Ultrasound-assisted extraction is also getting attention, where ultrasound waves create acoustic cavitation through the extraction medium by destroying the cell wall and releasing the cell contents [21].

Deep eutectic solvents (DESs) are also becoming popular in the extraction industry as viable substitutes for organic solvents, due to their safety and environmental friendliness [22].

Among all these methods, hydrodistillation is widely recognized as a standard technique for extraction of essential oils from floral biomass, gives a 1.66–1.83% yield [23], and is capable of extracting the majority of volatile compounds found in the aromatic resins, primarily located on the bracts surrounding the female flowers of the hemp plant [24]. Hydrodistillation is often chosen over other extraction methods because it is a simple, cost-effective, and greener because it only requires water for the extraction of essential oils, thus eliminating the need for organic solvents, high-pressure systems, or high-energy input used in steam distillation and supercritical CO_2_ extraction.

In a typical floral hemp variety processing scenario, the floral biomass constitutes approximately 12–21% of the total harvested plant material [25]. Although floral biomass after extraction is often treated as a by-product, this study demonstrates its value as a dual feedstock for both essential oil extraction and fiber production, contributing to higher overall material utilization. In addition to essential oil recovery, the residual floral biomass represents a largely unexplored lignocellulosic resource for fiber-based applications. Whereas hemp bast and hurd fibers from fiber hemp varieties have been extensively studied and utilized in pulping and papermaking, the potential of spent fibers from floral hemp varieties has received limited academic attention and is often overlooked as waste. Floral hemp residues still contain appreciable cellulose content and fibrous structure, suggesting their suitability as a secondary fiber source. Converting spent floral biomass into pulp fibers offers a sustainable pathway to valorize this residue, reduce waste generation, and serve as a partial substitute for conventional fibrous raw materials. From a papermaking perspective, such fibers may be particularly suited for lightweight paper grades, molded fiber products, or blended pulp systems where moderate mechanical performance is acceptable, thereby aligning with integrated hemp biorefinery and circular economy concepts.

In the present study, the essential oil was extracted from three floral hemp varieties by hydrodistillation. The qualitative and quantitative analysis of the chemical composition of hemp essential oils, such as the main volatile compounds, α-pinene, β-myrcene, α-humulene, and α-terpineol, was obtained using GC-MS. The spent floral biomass, which remained as the residue after hydrodistillation, was further converted into fiber through soda pulping, and the fiber properties were evaluated for papermaking.

## 2. Results and Discussion

### 2.1. GC-MS

The chemical profile of the essential oil of three different hemp varieties, Sour Space Candy (SSC_EO), Suver Haze 3N (SHN_EO), and Pinewalker 3N (PWN_EO), was investigated by GC-MS to analyze compounds of interest, such as terpenes, sesquiterpenes, and oxygenated compounds. The results of GC-MS are presented in Table 1 and Table 2, whereas the chromatograms of all essential oils are represented in Figure S1. Table 1 summarizes the qualitative and semi-quantitative analysis of the compounds identified in the essential oil samples, along with their average percentage peak areas, while Table 2 reports the concentrations (µg/mg) obtained from quantitative analysis.

Seventy-nine compounds were qualitatively identified using the MS spectral library (after background spectral subtraction), and calculated retention index (RI_c_) from a mixture of n-alkanes (C_8_–C_20_) in n-hexane, matched with the MS library, NIST Chemistry WebBook, and reported retention indices (RI_r_). A total of 30 compounds were identified from the mixed standard terpene A, B, and mixture of A and B (Figures S2–S4), of which 24 were detected in the samples and semi-qualitatively analyzed. The essential oil yield obtained by hydrodistillation ranged from 1.24 ± 0.03% to 1.86 ± 0.04% (*w*/*w*), depending on hemp variety, summarized in Table 3.

The essential oil profiles varied significantly among the three hemp varieties, with specific terpenes consistently enriched in each. The SSC_EO exhibited the highest level of sesquiterpenes, such as naphthalene, decahydro-4a-methyl-1-methylene-7-(1-methylethylidene)-, (4aR,8aS)- (8.88%), selina-3,7(11)-diene (8.28%), and α-humulene (7.13%). Similarly, SHN_EO showed elevated amounts of sesquiterpenes, such as α-humulene (12.67%), β-copaene (7.12%), and selina-3,7(11)-diene (5.95%), while PWN_EO exhibited the higher level of monoterpenes, such as β-myrcene (13.96%) and α-pinene (7.48%). SSC_EO and SHN_EO are dominated by sesquiterpenes, potentially providing anti-aging effects [26]. PWN_EO was rich in monoterpenes, which have been successful for aromatic and antimicrobial applications [27,28,29].

These findings underscore the influence of hemp variety on terpene composition and emphasize the importance of varietal selection in tailoring essential oil profiles for specific end uses. The concentration of main terpene compounds in essential oils were as follows: β-myrcene (107.31–31.50 µg/mg), (E)-β-caryophyllene (85.80–32.31 µg/mg), α-terpinolene (69.17–9.91 µg/mg), α-pinene (58.31–37.23 µg/mg), and linalool (58.12–29.37 µg/mg) which is consistent with previously reported compositions of hemp essential oils [7,20,30]. When compared with supercritical CO_2_ extraction reported in the literature [31], increasing extraction pressure shifts terpene profiles from monoterpene-dominated fractions (≈52% at 10 MPa) toward sesquiterpene-enriched extracts (≈52% at 14 MPa).

In another recent study [32], steam distillation, which is another conventional extraction method, extracted a greater concentration of monoterpenes (54%) compared to sesquiterpenes (44.2%). Collectively, these findings indicate that terpene composition in hemp essential oils is not only modulated by cultivar genotype, but is also strongly influenced by extraction method.

### 2.2. Compositional Analysis

The cellulose, hemicellulose, lignin, ash, and extractives content of the samples, as floral hemp before hydrodistillation, spent floral biomass after hydrodistillation, and spent floral fiber obtained after soda pulping, are represented by FH, SFB, and SFF, respectively, and listed in Table 4. All the varieties of floral hemp contained 9.22–9.43% cellulose, 13.99–14.46% hemicellulose, 19.69–20.26% lignin, 14.02–15.34% ash, and 40.92–42.64% extractives.

After essential oil extraction, some extractives were extracted, which reflects the reduced extractive content of the spent floral biomass in the range of 31.87–32.99%. The spent floral fiber represented comparatively lower lignin content (9.22–10.87%) as most lignin was removed through soda pulping. The higher ash content in floral biomass was likely due to the accumulation of inorganic elements such as silica, aluminum, magnesium, potassium, phosphorus, sodium, and iron, which have been commonly reported in hemp biomass [33,34]. The ash content in floral hemp before extraction was 13.7–21.1% and after extraction was 18.7–30.5% as previously reported [35]. In another study, 17.40% ash content was determined in the mixed flowers and leaves of hemp [34]. Kleinhenz et al. reported that hemp flower exhibited 14.1% ash content before extraction and 25.7% after extraction [36]. These findings are consistent with the present study, where high ash content was also observed across floral hemp, spent floral biomass, and spent floral fiber. High ash content is undesirable during pulping as it creates problems during the recovery of cooking liquor [37,38]. Additionally, increased ash content in biomass sometimes negatively impacts the strength of the fibers in composite applications [39]. Soil type, fertilizer type, fertilizer concentration, impurities, and other geological factors affect the ash content of the biomass in other biomaterials [33]. It has been shown that the floral materials of hemp uptake silica from the soil, which could be the reason behind the high ash content of the floral biomass [40]. However, ash levels can be partially controlled through pre-processing steps such as washing, mechanical cleaning, chemical leaching, and optimized pulping conditions, which have been reported to reduce inorganic contaminants in non-wood feedstocks [41,42].

Traditionally, hemp hurd contains 43% cellulose, 29% hemicellulose, 24.4% klason lignin, 1.4% ash, and 2.2% extractives [43]. Although the ash content of spent floral fibers is higher than that of conventional papermaking feedstocks such as hemp hurd, this does not necessarily limit industrial relevance. Materials with elevated ash content may still be advantageous for alternative valorization pathways, including soil amendment, biochar production, and circular biorefinery applications [40,44].

### 2.3. Fiber Quality Analysis

Fiber length, width, fines, curl index, and kink index of the spent floral fibers of three varieties, SSC_SFF, SHN_SFF, and PWN_SFF, are listed in Table 5. The properties of fiber hemp found in the literature [43] are also listed in Table 5. The fiber hemp that was reported in Table 5 is the fiber derived from hemp hurds by different pulping methods such as kraft pulping, carbonate pulping, and soda pulping. This type of fiber was used as a control because it represents a standard hemp hurd fiber commonly used for papermaking, providing a well-established benchmark for evaluating the properties of the spent floral fibers. The fiber length of the spent floral fiber (0.37–0.44 mm) was lower compared to fiber hemp (0.73 ± 0.056 mm) [43].

Studies show that longer fiber offers superior strength properties because longer fiber with more joints has a larger surface area for bonding and may thus form a stronger network than shorter fibers. However, the shorter fiber length and higher fines (39.95–43.25%) content of spent floral fiber has the advantage of producing nanocellulose biopolymers, which require lower energy consumption [45]. The hydrodistillation process employed for essential oil extraction inevitably influences the physical characteristics of the spent floral fibers. Exposure to hot water and prolonged thermal treatment during hydrodistillation can promote fiber fragmentation. As a result, the spent floral fibers exhibited relatively higher fines content, and shorter fiber length compared to conventional hemp hurd or bast fibers.

The curl index of the spent floral fibers was in the range of 0.09–0.13, which is higher than that of fiber hemp (0.06 ± 0.014). The higher curled fibers can have a low tensile index, but can have a high tear strength in paper production [46]. Conversely, the damaged fibers are represented by the kink index; a higher kink index indicates more damaged fibers, which will affect mechanical strength. Comparing the results of the kink index of fiber hemp with spent floral fibers, it is obvious that spent floral fiber will have comparatively lower strength than fiber hemp. As shown in the Supplementary Information (Table S1), one-way ANOVA revealed statistically significant differences among the three varieties of spent floral fibers for fiber length, width, fines, curl index, and kink index (*p* < 0.05), indicating that the floral hemp variety has a significant influence on fiber length, width, fines, curl index, and kink index.

### 2.4. Kappa Number

The kappa number is an industry-specific descriptor of the concentration of residual lignin in the spent floral fibers. A higher kappa number means the spent floral fibers contain a higher amount of residual lignin [47]. The kappa number of the spent floral fibers of three varieties, SSC_SFF, SHN_SFF, and PWN_SFF, was in the range of 72.15–76.06 and is represented in Table 6. The initial lignin content of the floral biomass was around 19.69–20.26%, which was reduced by 46–52% after the soda pulping. The spent floral fibers contained a high lignin content of around 9.22–10.87%, which is responsible for the high kappa number. Such elevated kappa values indicate incomplete delignification, which is expected due to the mild soda pulping conditions applied and the inherently high extractives and ash content of floral biomass. The kappa number of pulp fibers is strongly influenced by both raw material composition and pulping conditions, including alkali charge, cooking temperature, and reaction time. The kappa number of the pulp fibers also depends on the pulping conditions. Literature reports that hemp hurd fibers pulped with 15–19% active alkali exhibit kappa numbers in the range of 54 to 66, which is comparable with the present kappa numbers [47]. From a papermaking perspective, elevated kappa numbers are typically associated with lower brightness, reduced fiber flexibility, and diminished inter-fiber bonding, consistent with the lower mechanical properties and brightness observed for handsheets made exclusively from spent floral fibers.

### 2.5. Freeness

The pulp freeness is based on the principle of how fast the water from a diluted suspension of fiber with little surface area will drain through a specific screen plate. The drainability of the pulp suspension is the measure of freeness. The higher freeness means the drainability of the pulp is faster, and the lower means slower. The freeness value of the spent floral fibers of three varieties, SSC_SFF, SHN_SFF, and PWN_SFF, was in the range of 217–248 CSF and is reported in Table 6. The spent floral fibers had a higher amount of fines and shorter fiber lengths. In the presence of water, they swelled more. Due to having a high surface area and the presence of more cracked and broken fibers, they had higher water holding capacity, lower water drainability, and lower freeness. Low freeness values are typically associated with pulps containing high fines content and short fibers, which increase resistance to water flow during sheet formation. The relatively low freeness observed for spent floral fibers indicates slower drainage behavior, which can negatively affect papermaking runnability by increasing sheet forming time and energy demand during dewatering. Traditionally, hemp hurd fibers have a freeness in the range of 402–700 CSF, previously reported, due to having comparatively longer fiber length and lower fines content [48].

### 2.6. WRV

The WRV measurement of the spent floral hemp fibers indicates the total water holding capacity of a prescribed mass of fiber. Higher WRVs suggest greater fiber swelling and porosity, typically associated with shorter fiber length and increased fines content [49]. The WRV of the spent floral fibers of three varieties, SSC_SFF, SHN_SFF, and PWN_SFF, was in the range of 2.09–2.24 g/g and is reported in Table 6. The PWN_SFF exhibited the lowest WRV of 2.09 g/g and the SHN_SFF exhibited the highest WRV of 2.24 g/g, which indicates that fiber samples with high fines content and short fiber length have greater water absorption and expansion. This trend is consistent with the fiber quality analysis data, where SHN_SFF had the highest fines content and shorter average fiber length compared to the other varieties. The elevated WRV in such samples indicates a higher capacity to hold water within the fiber matrix, which can influence pulp processing characteristics such as freeness. Traditionally, hemp hurd fibers have a WRV in the range of 0.60–0.72 g/g, previously reported, which can be attributed to their longer fiber length and lower fines content, resulting in reduced water-holding capacity compared to more fiber cell wall damaged or fine-rich fibers [49,50].

### 2.7. Viscosity

Viscosity is a rheological flow rate determination of the degree of polymerization based on dissolving the lignin-free pulp in cupriethylenediamine [51]. Pulp viscosity is a good indicator of the strength of the fiber [51]. The viscosity of the obtained spent floral fibers of three varieties, SSC_SFF, SHN_SFF, and PWN_SFF, was low in the range of 3.67–3.98 cp and reported in Table 6. As the spent floral fibers contain higher amounts of fines and shorter fiber length compared to hemp hurds, the viscosity was low. The decrease in viscosity is also a result of lower cellulose content and higher ash content. Also, the pulping process depolymerizes hemicellulose, which contributes to a decrease in the molecular weight distribution, reflected by a lower viscosity [52]. Viscosity of hemp hurd fiber was observed as approximately 65.87 cp in previous literature which is comparatively higher than the present reported value [53].

### 2.8. Pulp Brightness

Brightness is an important quality indicator in the pulp and paper industry, especially for products like printing paper, tissue, and high-grade packaging, where visual appearance matters. The brightness of the spent floral fibers of three varieties, SSC_SFF, SHN_SFF, and PWN_SFF, was observed in the range of 6.22–7.10% and reported in Table 6. All the spent floral fibers were unbleached, having a comparatively high kappa number around 72–76. Due to the presence of lignin content, approximately 9.22–10.87%, the brightness of the fibers was low. Brightness of 29–44% was observed for the hemp pulp prepared by kraft and soda-anthraquinone pulping methods in recent literature due to significant amounts of lignin removal [53,54]. As presented in the Supplementary Information (Table S2), one-way ANOVA revealed statistically significant differences among samples for kappa number, viscosity, WRV, and freeness (*p* < 0.05), whereas no significant difference was observed for ISO brightness (*p* > 0.05).

### 2.9. FTIR Analysis

**Figure 1:**
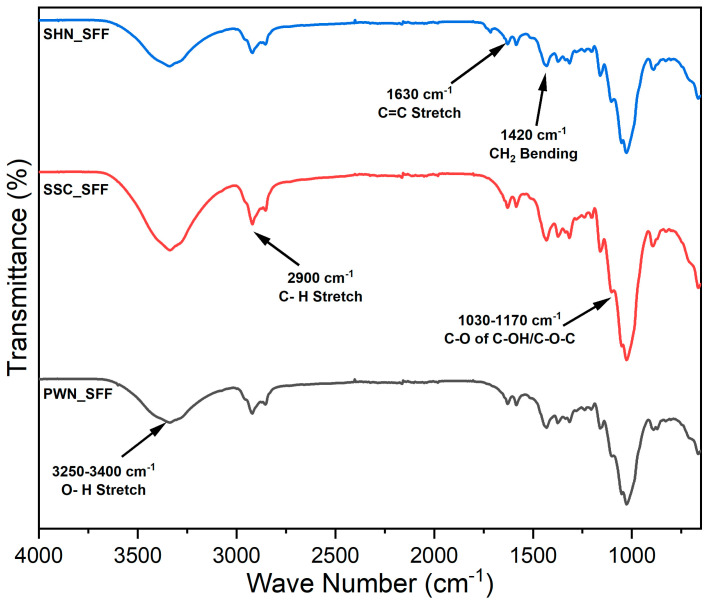
FTIR spectra of the spent floral fibers of three varieties, SSC_SFF, SHN_SFF, and PWN_SFF.

The FTIR spectra of the spent floral fibers of three varieties, SSC_SFF, SHN_SFF, and PWN_SFF, are represented in Figure 1. The appearance of the peak in the region of 1030–1170 cm^−1^ is due to C-O-C asymmetric stretching from ether linkages in polysaccharides and C-O stretching in primary and secondary alcohols in lignin-derived structures [55]. The bands at 1420, 1380, 1320 cm^−1^ are attributed to CH_2_ bending, C-H deformation of methyl groups, and skeletal or O-H bending vibrations, respectively, commonly arising from the structural features of cellulose, hemicellulose, and lignin [56]. The band near 1630 cm^−1^ is associated with C=C stretching in conjugated aromatic structures found in hemicellulose and lignin [43]. The band at 2900 cm^−1^ represents the presence of C-H stretching vibrations of cellulose. The broad O-H stretch around 3250–3400 cm^−1^ is a hallmark of cellulose and reflects its hydrogen-bonded network [57].

### 2.10. SEM Analysis

**Figure 2:**
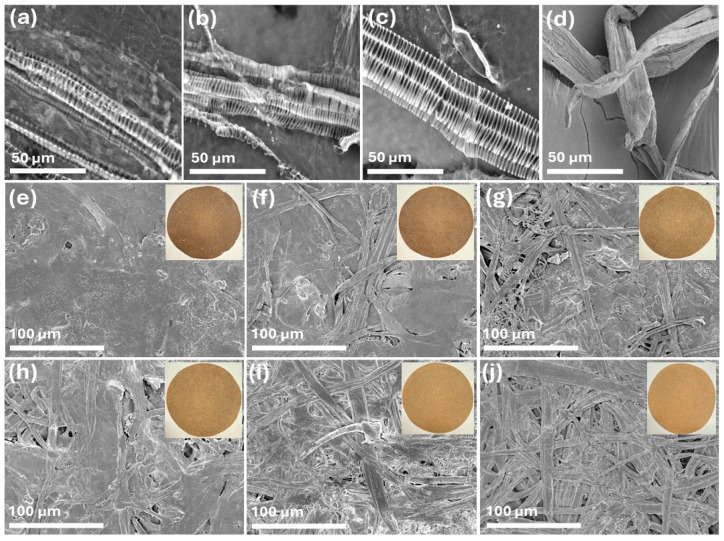
SEM images of spent floral fibers (**a**) SSC_SFF, (**b**) SHN_SFF, (**c**) PWN_SFF, and (**d**) hemp hurd fibers at 1 k× magnification and handsheets (**e**) FH, (**f**) 8FH2HH, (**g**) 6FH4HH, (**h**) 4FH6HH, (**i**) 2FH8HH, and (**j**) HH at 250× magnification.

The SEM images of spent floral fibers of three varieties, SSC_SFF, SHN_SFF, PWN_SFF, and hemp hurd fibers are represented in Figure 2a–d at 1 k× magnification. The spent floral fibers, after pulping, represented a unique morphology and exhibited a characteristic striated and elongated structure, with visible periodic banding along their length. The fibers maintained a generally aligned arrangement, though variations in surface texture and structural integrity were apparent. These observations suggest that the pulping process effectively separates the fibers while preserving their fundamental organization, making them potentially suitable for applications in biocomposites or paper production. The hemp hurd fibers in Figure 2d represent a flattened, ribbon-like structure [48]. The SEM images of the prepared handsheet FH, 8FH2HH, 6FH4HH, 4FH6HH, 2FH8HH, and HH are represented in Figure 2e–j at 250× magnification. The visual appearance of the handsheets is displayed as an inset in Figure 2e–j. The FH handsheet, which is made with 100% spent floral fiber, shows a smooth structure without any pores. The inset that represents the handsheet’s visual appearance shows a comparatively lower brightness, which was consistent with the ISO brightness values of 6.22–7.10%. The handsheet 8FH2HH, which was prepared with 80% spent floral fiber and 20% hemp hurd fibers, shows a slightly more heterogeneous and porous structure, with visible fiber bundles interwoven within the matrix. The surface roughness increased marginally, and the brightness improved compared to FH, indicating enhanced light reflectance due to the addition of lighter-colored hurd fibers. As the ratio of hemp hurd fibers increased from 40% to 100% (in samples 6FH4HH to HH), the SEM images revealed a progressive increase in surface porosity and fiber exposure. The fiber network became more open and interconnected, and the handsheets exhibited a lighter tone in the insets, indicating better brightness. The sample HH (100% hemp hurd fiber) shows the most fibrous and porous structure, suggesting reduced fiber packing density and the highest brightness.

### 2.11. Mechanical Properties

**Figure 3:**
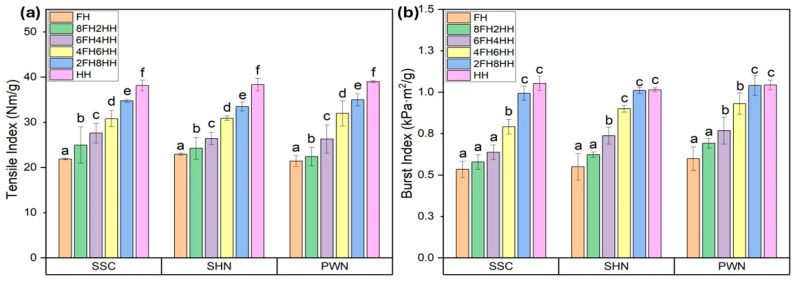
(**a**) Tensile index and (**b**) Burst index of FH, 8FH2HH, 6FH4HH, 4FH6HH, 2FH8HH, and HH handsheets made of a blend of spent floral fiber of three varieties of SSC, SHN, and PWN, and hemp hurd fiber. Values are reported as mean based on five independent handsheet measurements. Error bars represent standard deviation. Different lowercase letters above the bars indicate statistically significant differences among handsheets, as determined by one-way analysis of variance (ANOVA) followed by Tukey’s honestly significant difference (HSD) test (*p* < 0.05).

The mechanical properties, such as tensile strength and burst strength, of the handsheets made of spent floral fiber of three varieties, SSC, SHN, and PWN, are represented in Figure 3. The Figure demonstrates a clear improvement in both tensile (Figure 3a) and burst (Figure 3b) strength as the composition transitioned from 100% spent floral fiber to 100% hemp hurd fiber. The handsheets made entirely from spent floral fiber (FH) exhibited the lowest tensile (21.39–22.92 Nm/g) and burst (0.53–0.59 kPa·m^2^/g) indices across all three hemp varieties (SSC, SHN, and PWN), indicating weaker fiber bonding and limited mechanical integrity. As the proportion of hemp hurd fiber increased in handsheets, progressing through blends such as in 8FH2HH, 6FH4HH, 4FH6HH, 2FH8HH, HH, both tensile and burst indices showed a steady and significant rise by approximately 10%, 24%, 48%, 68%, and 79%. This improvement is attributed to the superior structural properties and bonding potential of hemp hurd fibers, which are richer in cellulose and have better fiber morphology compared to spent floral fibers.

The highest tensile index and burst index were observed for the 100% hemp hurd fiber handsheet (HH) in the range of 38.13–38.98 Nm/g and 1.01–1.05 kPa·m^2^/g. The measured properties of pulps and paper sheets produced from spent floral fibers were compared with those of commercially relevant hemp hurd fibers reported in previous literature. Lo et al. reported tensile index and burst index of hemp hurd handsheets in the range of approximately 48–74.8 Nm/g and 2.0–2.6 kPa·m^2^/g, respectively [58]. Tensile index around 42.4 Nm/g was reported for hemp stem fiber handsheets (including bast and hurd fibers) in another study [59]. The handsheet blends containing spent floral fibers in the present study exhibited slightly lower values, which can be attributed to the shorter fiber length, higher fines content, and elevated ash and lignin content in the spent floral fibers.

Despite these limitations, when spent floral fiber was combined with hemp hurd fiber at various ratios, such as 20–80%, 40–60%, or even up to 80–20%, the handsheets retained considerable mechanical strength, particularly when hemp hurd fiber was still present in modest amounts. For instance, handsheet blends like 6FH4HH or 4FH6HH achieved mechanical properties (tensile and burst index) approaching HH sheets, with only marginal trade-offs. This suggests that at least 40–60% of hemp hurd fiber content can be substituted with spent floral fiber without severely compromising paper strength or integrity. One-way ANOVA revealed a statistically significant effect of FH/HH ratio on tensile index and burst index for SSC, SHN, and PWN spent floral fiber samples (*p* < 0.05). Post hoc Tukey HSD analysis confirmed significant differences among all treatments within each material, with tensile and burst index increasing progressively with increasing HH content. The complete ANOVA summaries are provided in the Supplementary Information (Tables S3 and S4).

Beyond the handsheet evaluations performed in this study, the spent floral pulp fibers demonstrated potential for a broader range of value-added applications. Given their moderate mechanical performance, these fibers may be suitable for use in low-strength paper products, lightweight paper grades, molded fiber products (e.g., trays, cushioning materials), tissue, hygiene products, and biodegradable packaging applications. Blending spent floral pulp with reinforcing fibers, such as hemp hurd, recycled kraft pulp, or other lignocellulosic fibers could further enhance strength properties and expand suitability for paperboard and specialty paper products. In addition to papermaking, the pulp may also be explored as a reinforcing component in bio-based composites, biodegradable packaging inserts, or agricultural products such as seedling trays and mulch-based materials. These utilization pathways highlight the potential of spent floral biomass as a versatile feedstock within an integrated hemp biorefinery framework, aiming to maximize material utilization and reduce waste.

## 3. Materials and Methods

### 3.1. Materials

Three floral hemp varieties (Sour Space Candy, Suver Haze 3N, and Pinewalker 3N) were grown on the Mountain Horticultural Crops Research and Extension Center in Mills River, NC, USA. The cultivars were selected based on their agronomic suitability and chemical relevance to floral hemp production. The selected cultivars represented feminized, low-THC (<0.3%) floral hemp varieties capable of producing approximately 15–30% total cannabinoids under well-managed field conditions, with flowering times ranging from approximately 7 to 10 weeks after planting, depending on cultivar. Floral hemp cultivars were sown in a greenhouse and then grown in an outdoor field on Hayesville loam soil with 7–15% slopes. Prior to transplanting, the field was disked and tilled, and raised beds were formed and covered with white-on-black plastic mulch.

Drip irrigation lines were installed to provide supplemental irrigation as needed, delivering up to approximately 2 inches of water per week. Preplant soil amendments included lime, applied at a rate of 0.5 tons acre^−1^ and an organic granular fertilizer Holganix 7-9-5, purchased from AgCare Products (Candler, NC, USA), applied at 1050 lb acre^−1^. Feminized hemp seeds were sown by hand into cell trays filled with soilless growing media and propagated in a greenhouse for approximately 8 weeks prior to field transplanting. Beginning four weeks after germination, seedlings were fertilized weekly with Neptune’s Harvest fish and seaweed emulsion, purchased from Seven Springs Farm Supply (Check, VA, USA), at a dilution rate of 2 oz. per gallon of water.

Transplanting to the field occurred in June, with plants spaced approximately 5 ft apart within raised beds. Floral biomass was harvested in September at optimal maturity, determined based on trichome development and pistil characteristics, to maximize essential oil quality. All seeds used in this study were sourced from Oregon CBD Seeds (Independence, OR, USA). Sodium hydroxide (pellets) was purchased from Sigma Aldrich (Milwaukee, WI, USA). Reference standards, such as cannabis terpene mix A and B used for GC analysis, and n-hexane (HPLC grade), were also purchased from Sigma Aldrich (Milwaukee, WI, USA).

Commercial hemp essential oil was purchased from Amazon. Alkane standard solution (C_8_–C_20_) in n-hexane was purchased from Sigma Aldrich (Milwaukee, WI, USA). Hemp hurd fibers were developed in-house by the soda pulping method by using 14% NaOH (based on Na_2_O) at 160 °C for 3 h at a 1:8 hemp hurd to liquor ratio and used as is.

### 3.2. Methods

#### 3.2.1. Hydrodistillation

Extraction of essential oil was carried out by hydrodistillation using the fresh inflorescences of three different varieties in a Clevenger apparatus. 150 g of floral biomass was placed in a 3000 mL round-bottom flask with 1875 mL of water by maintaining a 1:15 floral biomass to water ratio. The flask was placed in a heating mantle and connected to the Clevenger apparatus and to a condenser. The mixture was boiled for 3 h. The hydrodistillation parameters were preliminarily optimized for each hemp variety by testing three floral biomass-to-water ratios (1:10, 1:15, and 1:20) and three extraction durations (1 h, 2 h, and 3 h). Among these, the 1:15 floral biomass-to-water ratio and 3 h duration at boiling temperature consistently resulted in the highest essential oil yield across all varieties. After finishing hydrodistillation, the water was removed from the bottom of the Clevenger apparatus, and light yellow-colored essential oil was collected in glass vials. The obtained essential oil samples were saved in the refrigerator at a controlled temperature of 4 °C in sealed amber glass vials for further characterization and analyzed within 7 days of collection. The essential oil samples were abbreviated as SSC_EO, SHN_EO, and PWN_EO according to the floral hemp variety name. The spent floral hemp biomass was squeezed to remove water and then air-dried without grinding prior to pulping. Air-drying was chosen as a gentle method to preserve fiber integrity and minimize hornification (irreversible fiber densification) that could occur with oven drying. Additionally, the biomass was used in its natural form without grinding to maintain the structural characteristics of the fibers and reduce energy input. The yield of essential oils and spent floral biomass is reported in Table 3.

#### 3.2.2. Soda Pulping of Spent Floral Biomass

Pulping of the residual spent floral biomass was carried out at 160 °C for 3 h at a 1:8 spent floral biomass to liquor ratio by using 14% NaOH (based on Na_2_O). After 3 h, the cooked spent floral biomass was washed with 2000 mL of water, and the black liquor was collected. The cooked spent floral biomass was disintegrated at 3000 rpm for 5 min to achieve the spent floral pulp fibers and saved for further characterization. The yield of spent floral pulp fibers is reported in Table 3. As shown in the Supplementary Information (Table S5), one-way ANOVA revealed statistically significant differences among the varieties for essential oil content, spent floral biomass, and spent floral fiber content (*p* < 0.05). Post hoc Tukey HSD analysis confirmed significant pairwise differences among all samples, indicating that the variety of floral hemp significantly influences essential oil retention and the distribution of spent floral biomass and fibers.

#### 3.2.3. Handsheet Making

**Figure 4:**
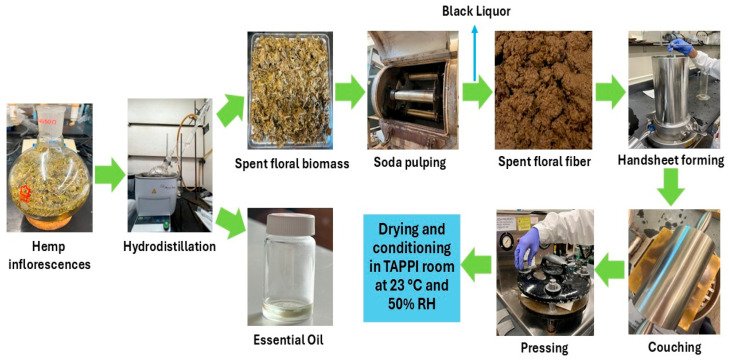
The hydrodistillation process of floral hemp biomass, soda pulping of residual spent floral biomass, and handsheet preparation steps from spent floral fibers.

The spent floral fibers were mixed with hemp hurd fibers at 0%, 20%, 40%, 60%, 80%, and 100% ratios and diluted to 0.3% consistency. Handsheets of 60 g/m^2^ basis weight were prepared following the TAPPI T 205 method by using a standard handsheet former (Testing Machines Inc., New Castle, DE, USA). After forming, the handsheets were pressed and dried overnight at 23 °C and 50% relative humidity (RH) by placing them into a drying ring before testing. The handsheets prepared from the spent floral fibers with hemp hurd fibers at 100–0%, 80–20%, 60–40%, 40–60%, 20–80%, and 0–100% ratios were abbreviated as FH, 8FH2HH, 6FH4HH, 4FH6HH, 2FH8HH, and HH, respectively. Figure 4 represents the hydrodistillation process of floral hemp biomass, soda pulping of residual spent floral biomass, and handsheet preparation steps from spent floral fibers.

### 3.3. Characterization

#### 3.3.1. GC-MS

Essential oil from three different varieties of floral hemp was analyzed by gas chromatography coupled with a mass spectrometer (GC/MS 9000/5977B, Agilent Technologies, Inc., Santa Clara, CA, USA) equipped with the capillary DB-5 column (30 m × 0.25 mm inner diameter (i.d.), 0.25 µm film thickness). The column temperature was programmed at 45 °C, then increased to 100 °C at the rate of 2 °C/min, then raised to 250 °C at 5 °C/min, again raised to 280 °C at 11 °C/min, and held for 15 min. The injection volume was 1 µL, with a split ratio of 1:20. Helium was used as the carrier gas, at a flow rate of 1.2 mL/min. The injector, transfer line, and ion-source temperatures were 250 °C, 280 °C, and 230 °C, respectively. MS detection was performed with electron ionization (EI) at 70 eV, operating in the full-scan acquisition mode in the *m*/*z* range 40–500. The essential oils and the reference standards were diluted 1:20 (*v*/*v*) with *n*-hexane before GC-MS analysis. Commercial hemp essential oil was used as a control sample. The *n*-alkane standard solutions (C_8_–C_20_) in n-hexane were injected on the same capillary column under the identical conditions for retention index (RI) calculation. The analysis was repeated three times with freshly prepared samples and standards.

#### 3.3.2. Compositional Analysis

The carbohydrates (cellulose and hemicellulose), total solid, ash, lignin, and extractive content in the FH, SFB, and SFF were determined and quantified following the standard test methods ASTM E1758-01, ASTM E1756-08, E1755-01, E1721-01, E1690-08 or the laboratory analytical procedure (LAP) (NREL/TP-510-42618) [60,61,62,63,64]. Briefly, cellulose was quantified by the amount of glucose determined through high-performance liquid chromatography (HPLC). Hemicellulose was measured by summing xylose, arabinose, mannose, and galactose subunits. The lignin content was determined by summing the acid-insoluble and soluble lignin, while the ash content corresponds to the residue remaining after dry oxidation. For the extractive’s determination, the FH, SFB, and SFF of three different varieties were air dried, milled, and sieved with a mesh no. 40 and analyzed using the Soxhlet and described here briefly. Approximately 3.0 g of the milled and sieved samples was weighed into pre-weighed extraction bags, placed inside a thimble, and then the thimble was inserted into the Soxhlet extractor. Approximately 350 mL of a toluene/ethanol mixture (2:1, *v*/*v*) was transferred into a clean 500 mL flask, and the extraction was allowed to run for 8 h. Then the extracting solvent was changed to ethanol, and the extraction continued for an additional 2 h after which the extraction bag containing the sample was placed in the oven at 45 °C overnight to dry. The extractives are calculated from the weight loss of the sample using Equation (1).

(1) $$\%\;Extractives=\frac{sample\;weight\;before\;extraction-sample\;weight\;after\;extraction}{sample\;weight\;before\;extraction}\times 100$$

#### 3.3.3. Fiber Quality Analysis

A high-resolution fiber quality analyzer (FQA 360 LDA17, OpTest Equipment Inc., ON, Canada) was employed to determine the fines in spent floral pulp fibers. The fibers were disintegrated with a British disintegrator for 5 min before analysis [65].

#### 3.3.4. Kappa Number

The lignin content of the spent floral fibers was measured by kappa test according to the Tappi T 236 om-99 method. Briefly, air-dried spent floral fiber samples were weighed according to the estimated kappa number, blended uniformly, and transferred to a 2 L beaker. 100 mL of 0.1 N KMnO_4_ and 100 mL of 4 N H_2_SO_4_ were mixed and added to the pulp solution in 2 L beaker. The pulp solution was then stirred for 10 min, the temperature was recorded, and 20 mL of 1 N KI was added at the end. Then, the mixture was titrated against 0.20 N Na_2_S_2_O_3_ using starch as an indicator. The same test was repeated three times to calculate the average kappa number following Equation (2), where *K* is the kappa number, *p* is the amount of 0.1 N permanganate actually consumed by the test specimen in ml, *f* is the correction factor, *w* is the weight of the moisture free spent floral fiber samples, and *t* is the temperature. A blank test following the same procedure was also performed without the spent floral fiber sample.

(2) $$K=\frac{pf}{w}\;\left[1+0.013\left(25-t\right)\right]$$

#### 3.3.5. Freeness

The freeness of the spent floral fibers was measured according to TAPPI T 227 using a freeness tester (Testing Machines Inc., New York, NY, USA). Briefly, a disintegrated 1000 mL pulp suspension of 0.3% consistency was poured into the chamber of the freeness tester, and the top lid was closed. The bottom lid was opened, and the discharge from the side orifice into the graduate cylinder was measured in mL. The values were reported in CSF (Canadian Standard Freeness) corrected to 0.3% consistency and 20 °C.

#### 3.3.6. Water Retention Value (WRV)

The WRV of the spent floral fibers was measured following the ISO 23714:2007 method using a centrifuge (Universal 320, Hettich Instruments, Beverly, MA, USA). Briefly, 1.2 g of never-dried spent floral fiber on oven-dried basis (OD) was disintegrated in 480 mL of water to obtain a pulp suspension of 0.25% consistency. The suspension was poured onto a 30 mm diameter glass Gooch crucible connected with a vacuum-assisted suction flask to get a 1700 g/m^2^ test pad. Then, the test pad was centrifuged at 2370 RCF for 20 min to remove excess water. After that, the test pad was dried in a laboratory oven at 105 °C ± 2 °C for 4 h. The weight of the test pad after centrifugation and after drying was measured, and the WRV was determined from the weight difference.

#### 3.3.7. Viscosity

The viscosity of the spent floral fibers was measured according to Tappi T 230 om-08. Briefly, 0.25 g oven-dried fibers were placed in a stomacher bag, and 25 mL DI water was added to it. The mixture was beaten for 60 s. Then, 25 mL of 1 M cupriethylenediamine (CED) solution was added to the mixture after 60 s and was beaten again for 120 s. Afterwards, 7.2 mL of the slurry was transferred into the viscometer and allowed for temperature equilibrium to 25 °C. The time was recorded for the slurry to drain from the top line of the viscometer to the bottom line, and viscosity was calculated and reported in centipoise (cp).

#### 3.3.8. Pulp Brightness

The brightness of the spent floral fibers was measured by an advanced color spectrophotometer (TX-ISO, Technidyne corporation, New Albany, IN, USA) following the ISO 2470 standard method. Handsheets of the spent floral fibers were prepared first, and then the brightness was directly measured.

#### 3.3.9. FTIR Analysis

The chemical composition of the spent floral fibers was investigated by the Fourier transform infrared spectrophotometer (Frontier FTIR, PerkinElmer, Inc., Waltham, MA, USA) equipped with a universal attenuated total reflectance (ATR) sampling accessory. Measurement was performed in the range of 4000–650 cm^−1^ wavenumber. Fibers were air-dried and directly scanned by the spectrophotometer.

#### 3.3.10. SEM Analysis

A field emission scanning electron microscope (SU8700, Hitachi High-Tech Corporation, Tokyo, Japan) was employed to analyze the surface morphology of the spent floral fibers and the handsheets with an accelerating voltage of 20 kV and 2 kV, respectively. Samples were placed on carbon tape attached with a stub and coated with AuPd before imaging.

#### 3.3.11. Mechanical Properties

Mechanical properties, such as tensile strength and burst strength, of the spent floral fiber handsheets were evaluated by following TAPPI T 494 and TAPPI T 403 om-97 method using a horizontal tensile tester (84–56, The TMI Group of Companies, Veenendaal, The Netherlands) and a BF Perkins Mullen tester (Model A, Standex Company, Chicopee, MA, USA). Samples were cut into 100 × 15 mm strips before testing by the tensile tester. All the tests were carried out at the TAPPI standard conditioning temperature of 23 °C and 50% RH. Tensile strength and burst strength were measured for five replicates, and the mean value was reported as tensile index and burst index, after dividing by the basis weight.

#### 3.3.12. Statistical Analysis

All experimental data are expressed as mean ± standard deviation. Statistical analyses were performed using one-way analysis of variance (ANOVA) to evaluate the effect of sample type on the measured properties. When a significant difference was detected, pairwise comparisons among group means were conducted using Tukey’s honestly significant difference (HSD) post hoc test. A confidence level of 95% was applied, and differences were considered statistically significant at *p* < 0.05.

## 4. Conclusions

This study demonstrates an approach for the valorization of hemp floral biomass through hydrodistillation followed by soda pulping. Essential oil yields from three floral hemp varieties, Sour Space Candy (SSC), Suver Haze 3N (SHN), and Pinewalker 3N (PWN), were 1.45%, 1.24%, and 1.86% (weight by weight), respectively. GC-MS analysis identified 30 major volatile compounds, with monoterpenes and sesquiterpenes dominating the profiles. Notably, β-myrcene was most concentrated in PWN_EO (107.31 µg/mg), caryophyllene peaked in SHN_EO (85.80 µg/mg), and α-pinene was highest in SSC_EO (51.84 µg/mg), underscoring the distinct aromatic and therapeutic potential of each variety’s essential oil.

The resulting spent floral fibers exhibited a kappa number of 72.15 to 76.05, viscosity of 3.67 to 3.98 centipoise, freeness of 217 to 248 Canadian Standard Freeness, WRV of 2.09–2.24 g/g, and ISO brightness of 6.22 to 7.10 percent. When used alone, these fibers demonstrated lower mechanical properties (tensile index approximately 22.06 Newton meter per gram and burst index approximately 0.56 kilopascal square meter per gram) compared to pure hemp hurd fibers (tensile index approximately 38.48 Newton meter per gram and burst index approximately 1.04 kilopascal square meter per gram). However, hybrid handsheets formed from blends such as 6FH4HH and 4FH6HH achieved average tensile indices of 26.77 and 31.22 Newton meter per gram, respectively, showing substantial mechanical improvements while reducing hemp hurd fiber usage by up to 60 percent.

Based on the observed pulp characteristics and handsheet performance, spent floral fibers are most suitable for low- to moderate-strength paper products, particularly when blended with reinforcing fibers such as hemp hurd. Potential applications include lightweight paper grades, molded fiber products, biodegradable packaging, seedling trays, and other functional papers where high brightness and strength are not critical requirements. Overall, this study demonstrates that integrating essential oil extraction with soda pulping enables effective utilization of floral hemp biomass, linking phytochemical recovery with fiber-based material production and strengthening the role of hemp within a circular and sustainable bioeconomy framework.

## Figures and Tables

**Table 1:** Qualitative and semi-quantitative analysis of the compounds identified in the essential oil samples, along with their average percentage peak areas.

S. No.	RT	Compound	RI_c_	RI_r_	Percentage Peak Area (%)		
					SSC_EO	SHN_EO	PWN_EO
1	8.684	α-pinene	936	939	5.85 ± 0.00	3.42 ± 0.02	7.48 ± 0.00
2	9.367	Camphene	952	951	0.18 ± 0.01	0.27 ± 0.00	0.44 ± 0.02
3	10.674	Sabinene or β-pinene	978	979	3.40 ± 0.01	1.59 ± 0.01	5.52 ± 0.01
4	11.367	β-myrcene	991	991	4.45 ± 0.01	6.21 ± 0.00	13.96 ± 0.00
5	12.136	α-phellandrene	1005	1005	-	0.17 ± 0.03	0.58 ± 0.02
6	12.242	δ-3-carene	1008	1010	0.26 ± 0.00	-	0.38 ± 0.01
7	12.732	α-terpinene	1017	1018	-	-	0.25 ± 0.01
8	13.155	p-cymene	1026	1023	1.74 ± 0.02	0.39 ± 0.02	1.07 ± 0.01
9	13.405	Limonene	1030	1029	1.78 ± 0.01	2.58 ± 0.01	5.58 ± 0.01
10	13.857	(Z)-β-ocimene	1039	1038	-	0.20 ± 0.01	0.28 ± 0.01
11	14.454	(E)-β-ocimene	1049	1050	-	1.01 ± 0.02	1.42 ± 0.02
12	15.060	γ-terpinene	1060	1060	-	0.14 ± 0.02	0.25 ± 0.01
13	16.665	α-terpinolene	1085	1079	0.36 ± 0.00	1.91 ± 0.01	4.77 ± 0.01
14	16.858	Fenchone	1088	1088	0.14 ± 0.00	0.29 ± 0.03	0.30 ± 0.01
15	17.002	p-cymenene	1090	1090	0.41 ± 0.00	-	0.35 ± 0.02
16	17.800	Linalool	1101	1099	1.47 ± 0.03	2.35 ± 0.01	2.09 ± 0.01
17	18.156	Unidentified	1107		0.09 ± 0.01	1.47 ± 0.01	1.34 ± 0.01
18	18.839	Fenchol	1119	1113	0.74 ± 0.03	-	-
19	19.195	trans-2-pinanol	1124	1121	0.21 ± 0.01	0.38 ± 0.01	0.35 ± 0.02
20	20.300	Camphor	1141	1144	0.44 ± 0.02	0.37 ± 0.00	0.43 ± 0.01
21	22.156	endo-borneol	1168	1157	0.94 ± 0.01	1.38 ± 0.01	1.33 ± 0.01
22	22.618	Menthol	1174	1169	0.33 ± 0.05	0.21 ± 0.02	0.29 ± 0.01
23	22.705	Terpinen-4-ol	1175	1182	0.37 ± 0.01	-	-
24	23.041	Unidentified	1180		0.23 ± 0.03	-	-
25	23.387	p-cymen-8-ol	1184	1181	6.15 ± 0.01	0.25 ± 0.01	0.85 ± 0.01
26	23.801	α-terpineol	1189	1189	1.54 ± 0.02	1.52 ± 0.02	1.37 ± 0.01
27	33.042	Ylangene	1361	1361	0.28 ± 0.03	0.36 ± 0.02	0.21 ± 0.02
28	33.292	α-cubebene	1367	1365	0.13 ± 0.01	-	-
29	33.975	Unidentified	1384		0.55 ± 0.03	0.17 ± 0.01	0.34 ± 0.01
30	34.975	(E)-β-caryophyllene	1411	1419	5.85 ± 0.01	3.89 ± 0.00	6.10 ± 0.01
31	35.148	β-copaene	1416	1432	-	7.12 ± 0.00	-
32	35.225	cis-α-bergamotene	1418	1412	-	0.16 ± 0.01	-
33	35.418	trans-α-bergamotene	1425	1434	2.42 ± 0.02	4.30 ± 0.01	1.96 ± 0.02
34	35.668	α-guaiene	1433	1440	-	0.16 ± 0.02	-
35	36.095	α-humulene	1447	1454	7.13 ± 0.00	12.67 ± 0.00	6.69 ± 0.01
36	36.225	Alloaromadendrene	1451	1460	0.62 ± 0.03	-	0.55 ± 0.01
37	36.639	cis-β-farnesene	1464	1461	0.23 ± 0.02	0.18 ± 0.01	0.19 ± 0.02
38	36.716	γ-muurolene	1466	1476	0.84 ± 0.01	0.56 ± 0.02	0.58 ± 0.02
39	36.841	α-muurolene	1470	1490	0.19 ± 0.02	0.38 ± 0.02	0.20 ± 0.02
40	36.956	Unidentified	1474		0.74 ± 0.01	1.03 ± 0.02	0.53 ± 0.02
41	37.149	β-selinene	1480	1478	1.36 ± 0.01	1.10 ± 0.01	1.03 ± 0.02
42	37.254	Eremophila-1(10),11-diene	1483	1486	0.52 ± 0.01	0.92 ± 0.01	0.44 ± 0.02
43	37.360	β-cis-guaiene	1487	1482	1.56 ± 0.02	0.93 ± 0.02	1.16 ± 0.01
44	37.418	Valencene	1488	1484	0.14 ± 0.02	0.14 ± 0.03	-
45	37.543	Guaioxide	1492	1491	-	0.28 ± 0.02	0.17 ± 0.02
46	37.591	α-selinene	1494	1494	0.34 ± 0.01	0.14 ± 0.02	1.92 ± 0.01
47	37.706	Bicyclogermacrene	1497	1495	-	1.12 ± 0.02	-
48	37.764	β-bisabolene	1499	1500	1.53 ± 0.02	2.85 ± 0.01	-
49	37.908	Viridiflorene	1504	1505	1.47 ± 0.01	1.43 ± 0.01	1.07 ± 0.01
50	38.024	δ-cadinene	1509	1519	3.07 ± 0.01	1.99 ± 0.02	1.84 ± 0.01
51	38.430	Nerolidol	1526	1528	-	2.19 ± 0.02	-
52	38.572	Unidentified	1530		-	3.88 ± 0.01	-
53	38.639	Naphthalene, decahydro-4a-methyl-1-methylene-7-(1-methylethylidene)-, (4aR,8aS)-	1533	1544	8.88 ± 0.00	-	7.62 ± 0.00
54	38.756	Selina-3,7(11)-diene	1537	1540	8.28 ± 0.00	5.95 ± 0.00	6.86 ± 0.00
55	38.937	Caryophyllene oxide	1544	1574	0.23 ± 0.02	-	-
56	39.283	β-nerolidol	1557	1550	1.05 ± 0.02	1.77 ± 0.01	0.79 ± 0.02
57	39.755	trans-nerolidol	1574	1565	-	0.99 ± 0.02	0.50 ± 0.02
58	39.841	Caryophyllene oxide	1577	1574	3.17 ± 0.01	-	-
59	39.966	ar-tumerol	1582	1584	0.79 ± 0.02	-	-
60	40.207	Guaiol	1591	1589	2.36 ± 0.01	3.67 ± 0.01	1.98 ± 0.02
61	40.437	Humulene-1,2-epoxide	1599	1599	0.69 ± 0.02	0.83 ± 0.02	0.37 ± 0.02
62	40.505	2-naphthalenemethanol, decahydro-α,α,4a-trimethyl-8-methylene-, [2R-(2α,4aα,8aβ)]-	1602	1600	1.50 ± 0.02	-	-
63	40.832	γ-eudesmol	1616	1618	2.32 ± 0.01	2.80 ± 0.01	1.50 ± 0.02
64	40.957	Unidentified	1621		0.29 ± 0.02	0.75 ± 0.02	0.32 ± 0.02
65	41.005	1-epi-cubenol	1623	1620	0.55 ± 0.02	-	-
66	41.082	Unidentified	1627		0.33 ± 0.01	0.36 ± 0.02	0.21 ± 0.02
67	41.178	Caryophylla-4(14),8(15)-dien-5-ol	1631	1631	0.41 ± 0.01	0.36 ± 0.02	0.16 ± 0.02
68	41.322	τ-cadinol	1637	1639	0.29 ± 0.02	0.34 ± 0.02	0.20 ± 0.02
69	41.428	τ-muurolol	1639	1638	0.91 ± 0.01	0.25 ± 0.02	0.16 ± 0.02
70	41.640	7-epi-γ-eudesmol	1641		2.03 ± 0.02	2.09 ± 0.02	1.14 ± 0.02
71	41.745	α-eudesmol	1650	1649	0.74 ± 0.01	0.26 ± 0.02	-
72	41.851	Unidentified	1655		0.96 ± 0.02	1.46 ± 0.01	0.71 ± 0.02
73	41.938	Guai-1(10)-en-11-ol	1659	1655	0.22 ± 0.01	-	-
74	42.370	Unidentified	1667		3.00 ± 0.01	3.75 ± 0.01	1.57 ± 0.01
75	42.630	α-bisabolol	1680	1668	0.18 ± 0.01	0.23 ± 0.02	0.15 ± 0.01
76	43.034	Eudesm-7(11)-en-4-ol	1691	1680	0.09 ± 0.02	-	-
77	44.457	α-vetivol	1733	1756	0.21 ± 0.02	-	-
78	44.736	Unidentified	1773	1869	0.19 ± 0.01	-	-
79	45.736	Unidentified	1785	1813	0.10 ± 0.02	-	-

SSC_EO: Sour Space Candy essential oil; SHN_EO: Suver Haze 3N essential oil; PWN_EO: Pinewalker 3N essential oil: RT: retention time; RI_c_: calculated retention index based on *n*-alkanes (C_8_–C_20_); RI_r_: reported retention index from literature. Values are reported as mean ± standard deviation based on three independent GC–MS analyses (*n* = 3).

**Table 2:** Concentrations (µg/mg) obtained from the quantitative analysis of the essential oil samples.

S. No.	RT	Compound	RI_c_	RI_r_	Concentration (µg/mg)		
					SSC_EO	SHN_EO	PWN_EO
1	8.645	α-pinene	935	930	51.84 ± 0.15	37.23 ± 0.14	58.31 ± 0.11
2	9.367	Camphene	952	946	2.93 ± 0.10	2.24 ± 0.03	4.67 ± 0.03
3	10.665	β-pinene	978	979	16.44 ± 0.06	9.05 ± 0.06	22.48 ± 0.01
4	11.367	β-myrcene	991	991	31.50 ± 0.03	62.07 ± 0.06	107.31 ± 0.06
5	12.251	(+)-3-carene	1008	1006	6.13 ± 0.60	-	8.33 ± 0.60
6	12.723	α-terpinene	1017	1018	-	-	5.31 ± 0.90
7	13.155	p-cymene	1026	1023	7.90 ± 0.13	1.97 ± 0.04	3.09 ± 0.03
8	13.396	Limonene	1030	1029	14.82 ± 0.01	23.42 ± 0.05	33.42 ± 0.05
9	15.079	γ-terpinene	1060	1060	-	2.39 ± 0.04	2.31 ± 0.03
10	16.665	α-terpinolene	1085	1079	9.91 ± 0.06	40.74 ± 0.08	69.17 ± 0.03
11	16.877	Fenchone	1088	1096	0.50 ± 0.01	1.45 ± 0.11	0.84 ± 0.00
12	17.762	Linalool	1100	1099	29.37 ± 0.36	58.12 ± 0.05	35.05 ± 0.03
13	18.839	Fenchol	1118	1113	5.91 ± 0.04	17.86 ± 0.09	10.84 ± 0.03
14	20.512	Camphor	1144	1144	1.70 ± 0.07	-	-
15	21.589	Isoborneol	1160	1157	-	-	-
16	22.176	endo-borneol	1168	1157	2.01 ± 0.01	7.28 ± 0.05	4.76 ± 0.01
17	22.637	Menthol	1174	1169	16.32 ± 0.15	11.67 ± 0.04	10.38 ± 0.01
18	23.734	α-terpineol	1188	1189	23.29 ± 0.01	27.77 ± 0.06	17.58 ± 0.07
19	26.003	β-citronellol	1224	1228	-	-	-
20	26.417	(+)-pulegone	1231	1237	-	-	-
21	27.532	2,6-octadien-1-ol, 3,7-dimethyl-	1250		-	-	-
22	33.610	Geranyl acetate	1374	1382	-	-	-
23	34.716	α-cedrene	1400	1411	-	-	-
24	34.850	(E)-β-caryophyllene	1405	1419	40.89 ± 0.06	85.80 ± 0.12	32.31 ± 0.02
25	36.043	α-humulene	1429	1454	3.93 ± 0.04	6.20 ± 0.00	-
26	38.380	Nerolidol	1524	1528	13.56 ± 0.09	6.89 ± 0.05	12.25 ± 0.04
27	39.216	β-nerolidol	1556	1550	5.02 ± 0.01	6.23 ± 0.08	2.89 ± 0.04
28	40.428	Humulene-1,2-epoxide	1602	1598	4.11 ± 0.9	3.61 ± 0.10	1.88 ± 0.04
29	41.572	β-eudesmol	1649	1649	5.61 ± 0.02	1.51 ± 0.07	-
30	42.332	α-bisabolol	1680	1684	2.28 ± 0.10	1.64 ± 0.08	11.22 ± 0.03

SSC_EO: Sour Space Candy essential oil; SHN_EO: Suver Haze 3N essential oil; PWN_EO: Pinewalker 3N essential oil; RT: Retention time; RI_c_: Calculated retention index; RI_r_: Reported retention index. Values are reported as mean ± standard deviation based on three independent GC–MS analyses (*n* = 3).

**Table 3:** Yield of essential oil, residual spent floral biomass, and spent floral fibers.

S. ID	Variety	Essential Oil (%) *w*/*w*	Spent Floral Biomass (%)	Spent Floral Fibers (%)
SSC	Sour Space Candy	1.45 ± 0.07 ^b^	78.74 ± 0.02 ^b^	55.23 ± 0.02 ^c^
SHN	Suver Haze 3N	1.24 ± 0.03 ^a^	76.83 ± 0.04 ^a^	37.07 ± 0.03 ^a^
PWN	Pinewalker 3N	1.86 ± 0.04 ^c^	79.67 ± 0.03 ^c^	47.63 ± 0.02 ^b^

Values are reported as mean ± standard deviation based on five independent measurements (*n* = 5). Different superscript letters within a column indicate statistically significant differences (one-way ANOVA followed by Tukey’s HSD test, *p* < 0.05).

**Table 4:** Cellulose, hemicellulose, lignin, ash, and extractives content of floral hemp, spent floral biomass, and spent floral fiber.

S. ID	Component	Cellulose (%)	Hemicellulose (%)	Lignin (%)	Ash (%)	Extractives (%)
SSC	FH	9.22 ± 1.13	14.46 ± 1.84	20.25 ± 2.13	14.02 ± 0.17	40.92 ± 0.01
SSC	SFB	17.51 ± 1.54	19.93 ± 1.14	18.95 ± 0.41	11.31 ± 0.14	31.87 ± 0.01
SSC	SFF	24.48 ± 0.51	14.9 ± 1.16	9.37 ± 1.33	31.39 ± 0.44	20.18 ± 0.01
SHN	FH	9.45 ± 0.4	14.28 ± 3.09	19.69 ± 1.28	15.34 ± 0.07	42.64 ± 0.01
SHN	SFB	15.63 ± 1.67	18.61 ± 1.18	21.26 ± 2.53	11.65 ± 0.41	32.55 ± 0.01
SHN	SFF	24.11 ± 0.36	15.52 ± 0.59	9.22 ± 0.5	28.96 ± 0.27	23.48 ± 0.01
PWN	FH	9.43 ± 0.68	13.99 ± 0.82	20.26 ± 0.53	14.64 ± 0.08	42.26 ± 0.01
PWN	SFB	16.73 ± 1.52	21.43 ± 2.21	16.89 ± 1.15	11.1 ± 0.26	32.99 ± 0.01
PWN	SFF	23.13 ± 0.28	17.18 ± 0.78	10.87 ± 0.76	27.33 ± 0.14	22.81 ± 0.01

FH: Floral hemp; SFB: Spent floral biomass; SFF: Spent floral fibers. Values are reported as mean ± standard deviation based on three independent measurements (*n* = 3).

**Table 5:** Fiber length, width, fines, curl index, and kink index of the spent floral fibers.

S. ID	Length (mm)	Width (µm)	Fines (%)	Curl Index	Kink Index (mm^−1^)
SSC_SFF	0.42 ± 0.014 ^b^	28.80 ± 0.424 ^a^	42.95 ± 0.212 ^b^	0.13 ± 0.028 ^b^	1.41 ± 0.035 ^c^
SHN_SFF	0.37 ± 0.013 ^a^	30.05 ± 0.919 ^b^	43.25 ± 0.354 ^c^	0.09 ± 0.017 ^a^	1.06 ± 0.049 ^a^
PWN_SFF	0.44 ± 0.015 ^b^	28.70 ± 0.424 ^a^	39.95 ± 1.202 ^a^	0.10 ± 0.011 ^b^	1.21 ± 0.141 ^b^
Fiber Hemp, from [43]	0.73 ± 0.056	-	5.61 ± 1.466	0.06 ± 0.014	0.67 ± 0.841

Values are reported as mean ± standard deviation based on five independent measurements (*n* = 5). Different superscript letters within a column indicate significant differences (Tukey’s test, *p* < 0.05).

**Table 6:** Kappa number, viscosity, freeness, water retention value (WRV), and ISO brightness of the spent floral fibers.

S. ID.	Kappa Number	Viscosity (cp)	Freeness (CSF)	WRV (g/g)	ISO Brightness (%)
SSC_SFF	73.85 ± 1.06 ^a^	3.67 ± 0.07 ^a^	227 ± 3.00 ^b^	2.16 ± 0.02 ^a^	6.22 ± 1.02 ^a^
SHN_SFF	72.15 ± 0.21 ^b^	3.98 ± 0.17 ^b^	217 ± 5.00 ^a^	2.24 ± 0.05 ^b^	6.87 ± 0.28 ^a^
PWN_SFF	76.05 ± 0.77 ^c^	3.95 ± 0.02 ^b^	248 ± 3.24 ^c^	2.09 ± 0.09 ^a^	7.10 ± 0.17 ^a^

WRV: Water Retention Value. Values are reported as mean ± standard deviation based on five independent measurements (*n* = 5). Different superscript letters within a column indicate significant differences (Tukey’s test, *p* < 0.05).

## Data Availability

The data presented in this study are available on request.

## Supplementary Materials

The following supporting information can be downloaded at: https://www.mdpi.com/article/10.3390/molecules31030500/s1, Figure S1: GC-MS chromatograms of hemp essential oils from three different varieties, SSC_EO, SHN_EO, and PWN_EO, where the peaks correspond to the compound numbers listed in Table 1; Figure S2: GC-MS chromatograms of Terpene A standard; Figure S3: GC-MS chromatograms of Terpene B standard; Figure S4: GC-MS chromatograms of Terpene A and B mix standard; Table S1: One-way analysis of variance (ANOVA) for fiber length, width, fines, curl index, and kink index for spent floral fibers of three varieties, SSC_SFF, SHN_SFF, and PWN_SFF; Table S2: One-way analysis of variance (ANOVA) for kappa number, viscosity, freeness, WRV, and ISO brightness of spent floral fibers of three varieties, SSC_SFF, SHN_SFF, and PWN_SFF; Table S3: One-way ANOVA evaluating the effect of FH/HH ratio on tensile index of SSC, SHN, and PWN handsheets; Table S4: One-way ANOVA evaluating the effect of FH/HH ratio on burst index of SSC, SHN, and PWN handsheets; Table S5: One-way analysis of variance (ANOVA) for essential oil, residual spent floral biomass, and spent floral fibers of three varieties, SSC_SFF, SHN_SFF, and PWN_SFF.
