# Supplementary material for: Hydrodistillation-Based Essential Oil Extraction and Soda Pulping of Spent Hemp Biomass for Sustainable Fiber Production
Source: Molecules. 2026 Jan 31;31(3):500. doi: 10.3390/molecules31030500 (PMC12899041; doi:10.3390/molecules31030500)
Supplement: Supplementary file 1 [file molecules-31-00500-s001.zip › molecules-4068570-supplementary.pdf]

Supplementary Information

Hydrodistillation-Based Essential Oil Extraction and Soda Pulping of Spent Hemp Biomass for Sustainable Fiber Production

Munmun Basak [1], Stephen C. Agwuncha [1], Sharmita Bera [1], Margaret Bloomquist [2], Jeanine Davis [2], Lucian Lucia [1], and Lokendra Pal [1,*]

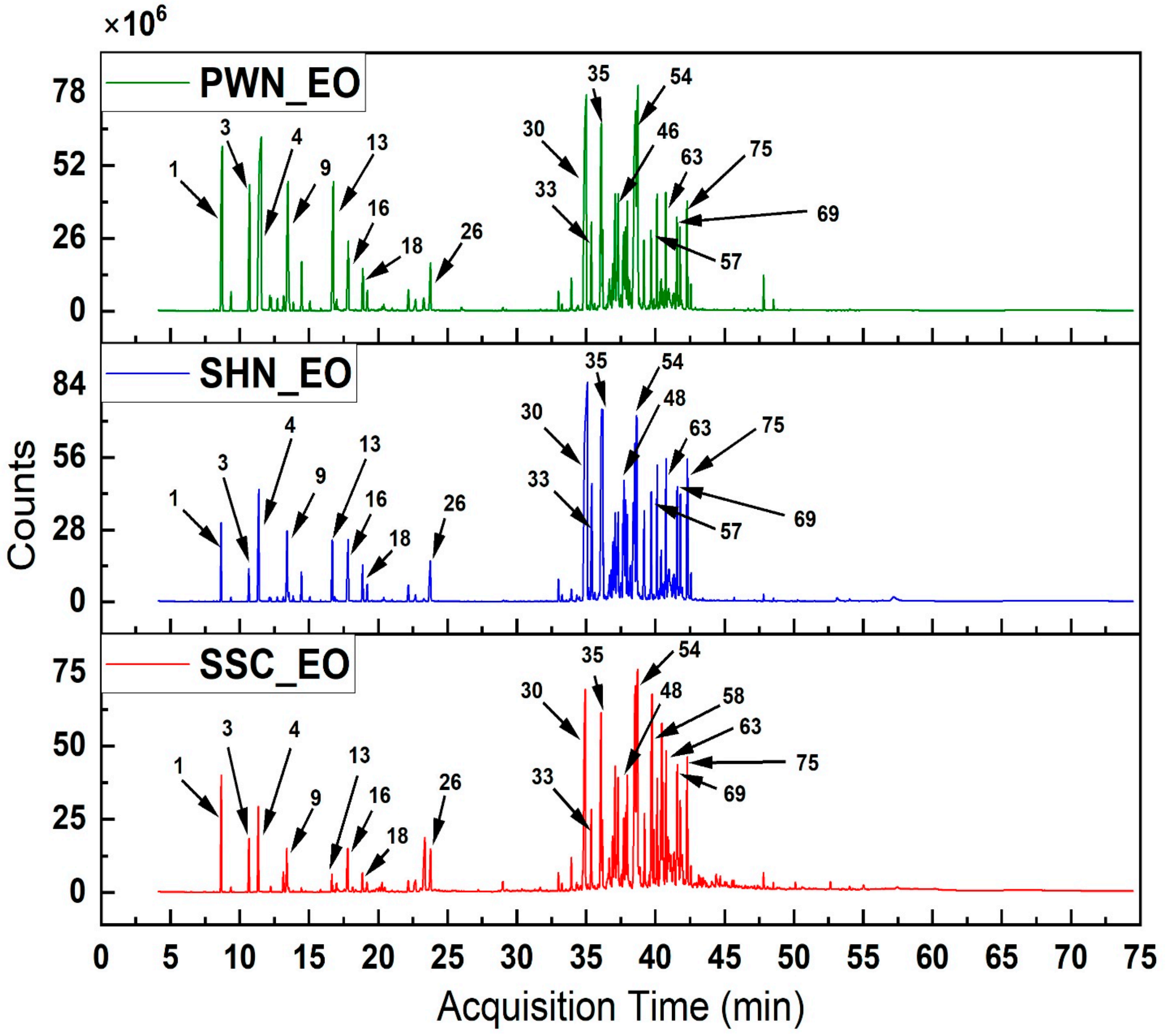

Figure S1. GC-MS chromatograms of hemp essential oils from three different varieties, SSC_EO, SHN_EO, and PWN_EO, where the peaks correspond to the compound numbers listed in Table 1.

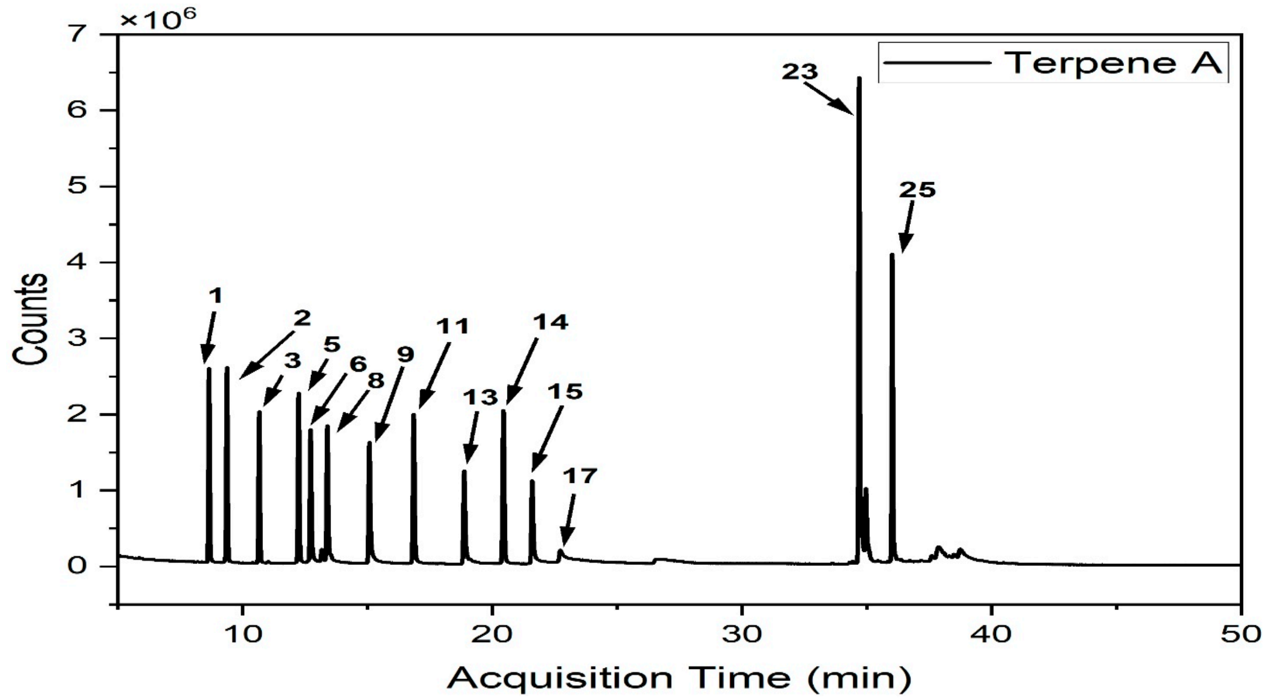

Figure S2. GC-MS chromatograms of Terpene A standard

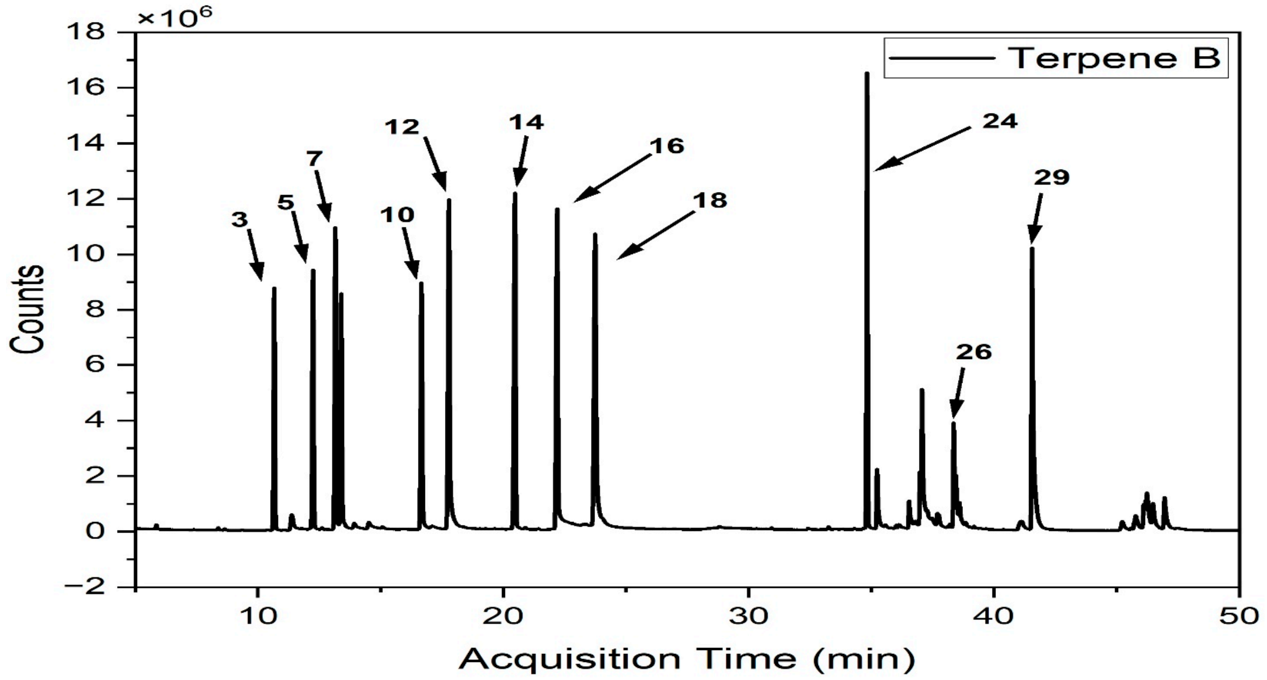

Figure S3. GC-MS chromatograms of Terpene B standard

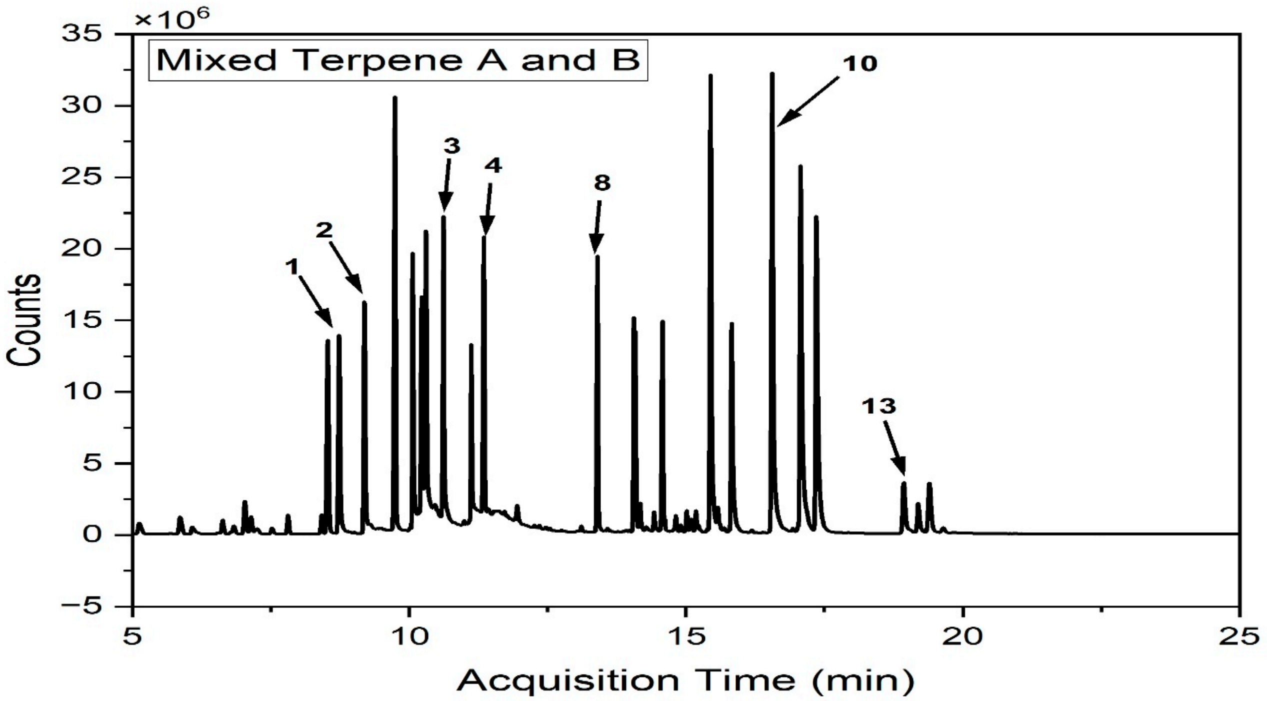

Figure S4. GC-MS chromatograms of Terpene A and B mix standard

Table S1. One-way analysis of variance (ANOVA) for fiber length, width, fines, curl index, and kink index for spent floral fibers of three varieties, SSC_SFF, SHN_SFF, and PWN_SFF

Properties	Source	SS	df	MS	F	*p*
Fiber Length	Between groups	0.0123	2	0.00615	29.28	<0.001
	Residual	0.00252	12	0.00021		
	Total	0.01482	14			
Width	Between groups	4.544	2	2.272	7.04	0.009
	Residual	3.876	12	0.323		
	Total	8.42	14			
Fines	Between groups	29.65	2	14.83	30.94	<0.001
	Residual	5.75	12	0.48		
	Total	35.4	14			
Curl	Between groups	0.00656	2	0.00328	6.04	0.015
	Residual	0.00651	12	0.00054		
	Total	0.01307	14			
Kink	Between groups	0.309	2	0.1545	19.47	<0.001
	Residual	0.095	12	0.0079		
	Total	0.404	14			

SS: Sum of squares; df: degrees of freedom; MS: Mean square. Statistical significance was evaluated at $p < 0.05$.

Table S2. One-way analysis of variance (ANOVA) for kappa number, viscosity, freeness, WRV, and ISO brightness of spent floral fibers of three varieties, SSC_SFF, SHN_SFF, and PWN_SFF

Properties	Source	SS	df	MS	F	*p*
Kappa Number	Between groups	38.2333	2	19.1167	32.52	<0.001
	Residual	7.055	12	0.5879		
	Total	45.2883	14			
Viscosity	Between groups	0.2971	2	0.1485	12.53	0.001
	Residual	0.1422	12	0.0119		
	Total	0.4393	14			
Freeness	Between groups	2503.33	2	1251.67	84.38	<0.001
	Residual	178	12	14.83		
	Total	2681.33	14			
WRV	Between groups	0.0612	2	0.0306	9.2	0.0038
	Residual	0.0399	12	0.0033		
	Total	0.1012	14			
ISO Brightness	Between groups	2.083	2	1.0415	2.72	0.106
	Residual	4.5908	12	0.3826		
	Total	6.6738	14			

SS: Sum of squares; df: degrees of freedom; MS: Mean square. Statistical significance was evaluated at $p < 0.05$.

Table S3. One-way ANOVA evaluating the effect of FH/HH ratio on tensile index of SSC, SHN, and PWN handsheets

Variety	Source	SS	df	MS	F	*p*
SSC	Between groups	929.08	5	185.82	21.65	< 0.001
	Residual	205.97	24	8.58		
	Total	1135.05	29			
SHN	Between groups	880.37	5	176.07	48.57	< 0.001
	Residual	87.01	24	3.63		
	Total	967.38	29			
PWN	Between groups	1262.95	5	252.59	30	< 0.001
	Residual	202.05	24	8.42		
	Total	1465	29			

SS: Sum of squares; df: degrees of freedom; MS: Mean square. Statistical significance was evaluated at $p < 0.05$.

Table S4. One-way ANOVA evaluating the effect of FH/HH ratio on burst index of SSC, SHN, and PWN handsheets

Variety	Source	SS	df	MS	F	*p*
SSC	Between groups	1.2001	5	0.24	60.38	< 0.001
	Residual	0.0954	24	0.004		
	Total	1.2955	29			
SHN	Between groups	0.9896	5	0.1979	57.02	< 0.001
	Residual	0.0833	24	0.0035		
	Total	1.0729	29			
PWN	Between groups	0.8769	5	0.1754	24.92	< 0.001
	Residual	0.1689	24	0.007		
	Total	1.0458	29			

SS: Sum of squares; df: degrees of freedom; MS: Mean square. Statistical significance was evaluated at $p < 0.05$.

Table S5. One-way analysis of variance (ANOVA) for essential oil, residual spent floral biomass, and spent floral fibers of three varieties, SSC_SFF, SHN_SFF, and PWN_SFF

Properties	Source	SS	df	MS	F	*p*
Essential Oil (%) w/w	Between groups	1.1076	2	0.5538	201.55	< 0.001
	Residual	0.033	12	0.00275		
	Total	1.1406	14			
Spent floral biomass (%)	Between groups	21.715	2	10.8575	10843.06	< 0.001
	Residual	0.012	12	0.001		
	Total	21.727	14			
Spent floral fibers (%)	Between groups	333.51	2	166.76	718954.2	< 0.001
	Residual	0.0028	12	0.00023		
	Total	333.51	14			

SS: Sum of squares; df: degrees of freedom; MS: Mean square. Statistical significance was evaluated at $p < 0.05$.
